# In Situ SAXS Measurement and Molecular Dynamics Simulation of Magnetic Alignment of Hexagonal LLC Nanostructures

**DOI:** 10.3390/membranes8040123

**Published:** 2018-12-02

**Authors:** Weiwei Cong, Weimin Gao, Christopher J. Garvey, Ludovic F. Dumée, Juan Zhang, Ben Kent, Guang Wang, Fenghua She, Lingxue Kong

**Affiliations:** 1 Institute for Frontier Materials, Deakin University, Geelong, Locked Bag 20000, Geelong, VIC 3220, Australia; weiwei.cong@deakin.edu.au (W.C.); weimin.gao@deakin.edu.au (W.G.); ludovic.dumee@deakin.edu.au (L.F.D.); jane.zhang@deakin.edu.au (J.Z.); guang.w@deakin.edu.au (G.W.); mary.she@deakin.edu.au (F.S.); 2 College of Metallurgy and Energy, North China University of Science and Technology, Tangshan 063009, China; 3 Australian Nuclear Science and Technology Organisation, Locked Bag 2001, Kirrawee DC, NSW 2232, Australia; chris.garvey@ansto.gov.au; 4 Institute for Soft Matter and Functional Materials, Helmholtz Zentrum Berlin, Hahn-Meitner-Platz 1, D-14109 Berlin, Germany; ben.kent@helmholtz-berlin.de

**Keywords:** alignment, magnetic field, hexagonal, lyotropic liquid crystals, nanofiltration, in situ small angle X-ray scattering (SAXS)

## Abstract

The alignment of nanostructures in materials such as lyotropic liquid crystal (LLC) templated materials has the potential to significantly improve their performances. However, accurately characterising and quantifying the alignment of such fine structures remains very challenging. In situ small angle X-ray scattering (SAXS) and molecular dynamics were employed for the first time to understand the hexagonal LLC alignment process with magnetic nanoparticles under a magnetic field. The enhanced alignment has been illustrated from the distribution of azimuthal intensity in the samples exposed to magnetic field. Molecular dynamics simulations reveal the relationship between the imposed force of the magnetic nanoparticles under magnetic field and the force transferred to the LLC cylinders which leads to the LLC alignment. The combinational study with experimental measurement and computational simulation will enable the development and control of nanostructures in novel materials for various applications.

## 1. Introduction

The alignment of nanostructures is of increasing research interest and importance to applications as ordered nanostructures will provide unprecedented benefits in tissue engineering [1,2], drug delivery [3,4], gas permeation [5], and water purification [6,7]. Lyotropic liquid crystals (LLCs) can self-assemble and form unique continuous hexagonal nanostructures that can be used as templates to synthesize organic [8,9] and inorganic [8,10] nanomaterials of distinctive properties. Although this process is a promising route for the fabrication of large quantities of low cost nano-structured and ultra-thin materials, the lack of long-range order in the template structure prevents the formation of highly anisotropic hexagonal structures. Isoporous membranes are highly demanded as these membranes with well-defined pore size and shape have numerous potential applications in areas such as micro- and nano-filtrations and gas separation. For LLC templated polymer membranes designed for nano-filtration (NF) applications, minor defects within the membrane materials will lead to the sharp decrease in selectivity. Therefore, slight variations in the orientation of the self-assembled domains may lead to localized disorder and loss in membrane separation performance [7,11]. It is critical to develop new strategies to produce stable, high density, and uniformly oriented cylindrical LLC nanostructures.

The application of an external force field, such as a magnetic [12,13] or an electric [14,15] field, can align LLC structures to produce anisotropic templates. Although alkyl chains in many of the surfactants possess a negative diamagnetic susceptibility and in anisotropic LLC phases, they can be aligned with magnetic field [16], particularly at very high magnetic fields (>3 T) as the magnetic susceptibility is very low [12,13]. It has been reported recently that a low magnetic field (<100 mT) could successfully align the lamellar or hexagonal phases when they were doped with magnetic nanoparticles [17,18,19]. However, no data and methods have been reported for in situ measurement of the alignment of LLC phases in low magnetic fields, as this dynamic process happens in a lyotropic liquid crystals, a procedure that is difficult to monitor in real-time with conventional characterisation techniques. 

Small angle X-ray scattering (SAXS) is used in a high-throughput manner for in situ characterization [20] of nanostructures of bulk long range order [21,22,23]. It allows for the determination of alignment and the identification of the self-assembled phases while molecular dynamics (MD) has shown its unique advantages in understanding the structure of membrane and the interfacial affinity [24]. The intermolecular interaction and force transport properties obtained from MD simulation are useful information in understanding alignment mechanism.

In this paper, the doped LLC nanostructure with magnetic nanoparticles was aligned under a magnetic field and analysed in situ by SAXS at the Australian Synchrotron. Molecular dynamics (MD) simulations were conducted to reveal the relationship between the imposed force of the magnetic nanoparticles under magnetic field and the force transferred to the LLC cylinders which leads to the LLC alignment. 

## 2. Materials and Methods

### 2.1. Materials

Polyethylene glycol diacrylate (PEGDA) (Formula, *n* = 11, MW = 575 g/mol), dodecyltrimethylammonium bromide (DTAB) (99%), 2-hydroxy-2-methylpropiophenone (HMPP) (97%), and magnetic nanoparticles Fe_3_O_4_ (average particle size of 5 nm, 5 mg/mL in water) were purchased from Sigma-Aldrich (St. Louis, MO, USA), and all chemicals were used as received.

### 2.2. Preparation of Liquid Samples Doped with Magnetic Nanoparticles

The lyotropic liquid crystalline system was prepared by mixing surfactant (DTAB) with the mixture of monomer (PEGDA), magnetic nanoparticles in water, and photo-initiator (HMPP), methanol (100:97:45.5:0.97:28.8 by weight). Methanol was used as solvent before it was removed by evaporation from liquid samples. The prepared mixture was made macroscopically homogeneous via vortex stirring and water bath (55 °C) for 24 h to reach equilibrium before further action of alignment under magnetic field was taken.

### 2.3. Magnetic Alignment

**Figure 1:**
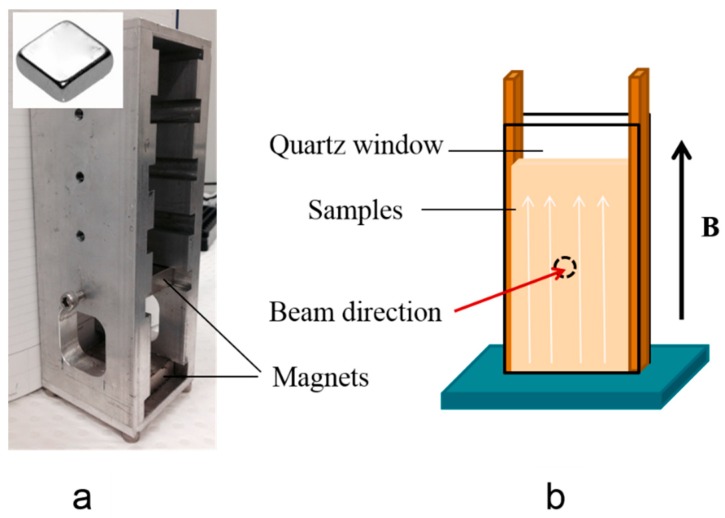
(**a**) The aluminium frame, used to position the two magnets; and (**b**) the cell used for in situ SAXS measurement.

The magnetic field was achieved from two neodymium blocks (50 mm × 50 mm × 12.5 mm, pull force: 42.9 kg, from AMF Magnetics, Rozelle, Australia) with a controllable distance to provide different field strengths (Figure 1a). 

A cell for holding liquid samples was developed by using parallel quartz windows to minimize X-ray adsorption and background scattering signal, with a distance of 2 mm between the windows to allow defining the path length of X-ray through the sample. The quartz windows and spacer were glued together around the sample using Araldite epoxy resin (RS, Northamptonshire, UK). This cell allowed the beam to horizontally pass through the sample that was orthogonal to the magnetic field direction (Figure 1b).

The alignment of prepared liquid samples was carried out under a magnetic field of 60 mT with in situ SAXS measurement. The samples at 55 °C were added to the homemade cell (Figure 1b), in a vertically-oriented magnetic field (60 mT). They then heated with an electric heat gun (50–60 °C, 2000 W, Bosch, Stuttgart, Germany) to change the sample from hexagonal to isotropic micellar phase [25]. The temperature was measured with a K thermocouple. The in situ SAXS measurements were started upon the cooling of the samples from 50–60 °C to ambient temperature.

The retention of the aligned structures in the resulting polymerised membranes was also studied. To prepare polymerized membranes with aligned structures, liquid samples confined between two glass slides at 55 °C (±3 °C) were placed in a magnetic field (0, 5, 17, and 60 mT). The magnetically-aligned samples were polymerized under a 300–400 nm UV light source (intensity 2.0 mW/cm^2^) in nitrogen atmosphere for 20 min. The polymerised membranes collected from the slides were sealed in thin Kapton layers for SAXS measurement to avoid further water evaporation during the measurement.

### 2.4. Characterization Techniques

A polarized light optical microscope (Olympus DP71, Olympus, Tokyo, Japan) equipped with a CCD camera in transmission mode was utilized to observe the phase behaviour of the as-prepared samples under 50× magnification.

In situ SAXS measurement was performed at the Australian Synchrotron on the SAXS/WAXS beamline over a range of scattering vectors, *q*, 0.5 nm^−1^ to 8.5 nm^−1^. *q* is defined as:

$$q=\frac{4\pi \sin\left(\theta /2\right)}{\lambda }$$

where *θ* is the scattering angle, *λ* is the X-ray wavelength. A wavelength of 0.127 nm and the sample to detector distance of 0.72 m were used. The scattering geometry defining the 2D diffraction pattern was recorded on a Pilatus 1M detector. The 2D data was reduced into 1D intensity versus q and intensity versus azimuthal angle for analysis using Fit2d (European Synchrotron Radiation Facility, Grenoble, France) [26], after subtracting the background from an empty cell in transmission and masking out the beamstop area. The acquisition time for each SAXS pattern was 1 s. These SAXS settings were mainly for measuring the alignment of the LLC nanostructure.

### 2.5. Simulation

The respective interactions of surfactant, DTAB, and monomer PEGDA with magnetic nanoparticles were simulated. As the maximum lengths of both DTAB and PEGDA molecules are much smaller than the size of magnetic nanoparticles, the simulations were performed for their interaction with a magnetite layer. The surface and monomer system were firstly optimized to obtain the interaction features and energy, thereafter, a quenching method was used in a constant particle number, volume and energy ensemble (NVE) with a quenching temperature of 450 K to obtain the interaction energy profile with interaction distance. The DTAB cylinder was approached by 50 ns dynamic simulation of a preassembled cylindrical micelle of 60 DTAB molecules [27]. In the simulations, a cutoff radius of 12 Å for a time step of 1 fs were applied. The simulations were carried out using the software package NAMD [28] and the CHARMM force field [29] was used.

## 3. Results

### 3.1. Alignment with Magnetic Field and Characterisation with Australian Synchrotron SAXS

**Figure 2:**
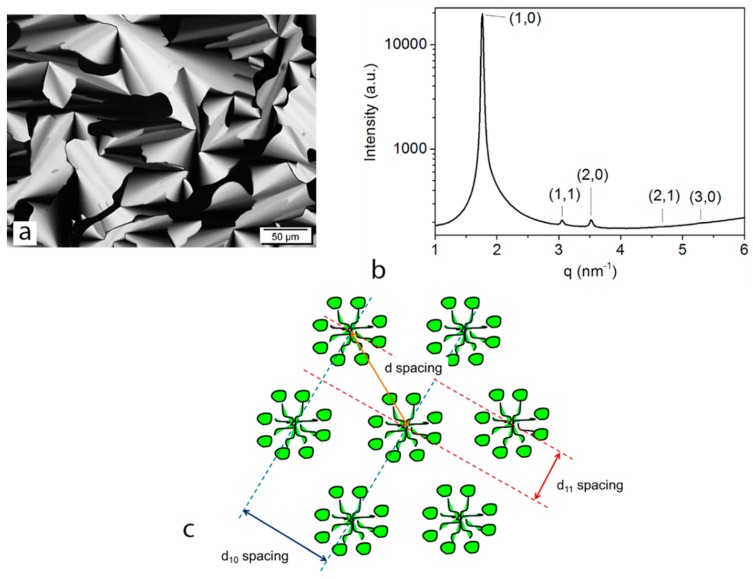
Hexagonal LLC phase formed using DTAB: (**a**) Polarized light microscopy image of the hexagonal phase; (**b**) SAXS profile of the hexagonal phase and indexing of each peak according to the hexagonal lattice; and (**c**) a schematic of the hexagonal phase including the origin of the important Bragg peaks.

Dodecyltrimethylammonium bromide (DTAB), an anionic surfactant dispersed in water, was used to form the hexagonal LLC structures [30]. A hydrophilic monomer, polyethylene glycol diacrylate (PEGDA), was then added and cross-linked within the aligned cylindrical nanostructure. The lyotropic liquid crystalline system was prepared by mixing surfactant (DTAB) with the mixture of monomer (PEGDA), magnetic nanoparticles in water, and photo-initiator (HMPP), methanol. A typical birefringence pattern, characteristic of hexagonal phase, can be found in the polarized optical microscopy (POM) image (Figure 2a).

A typical one-dimensional scattering pattern for the DTAB-Water sample collected from the Australian Synchrotron SAXS beamtime is shown in Figure 2b. The phase of this LLC was assigned according to the characteristic spacing of Bragg reflections. The hexagonal phase was indexed according to relationship between peak positions [31]:

$$q_{hk}=q_{10}\sqrt{h^{2}+hk+k^{2}}$$

The *d*-spacings belonging to particular crystallographic planes are shown in the schematic of hexagonal phase in Figure 2c. 

The spacing between two cylinders in the periodic arrangement can be calculated as follows:

(1) $$d=2D_{11}=\frac{D_{10}}{\sin60^{{}^{\circ}}}=\frac{2\pi }{q_{10}\times \sin60{}^{\circ}}=4.1\;n$$

**Figure 3:**
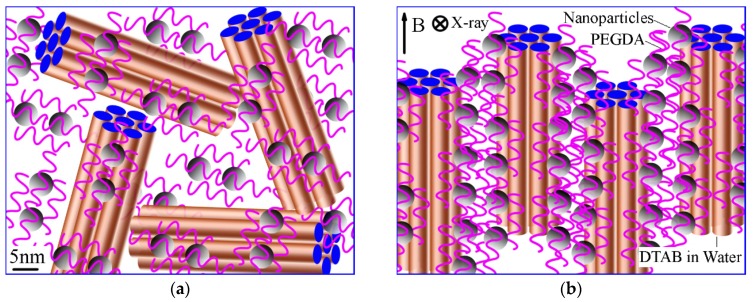

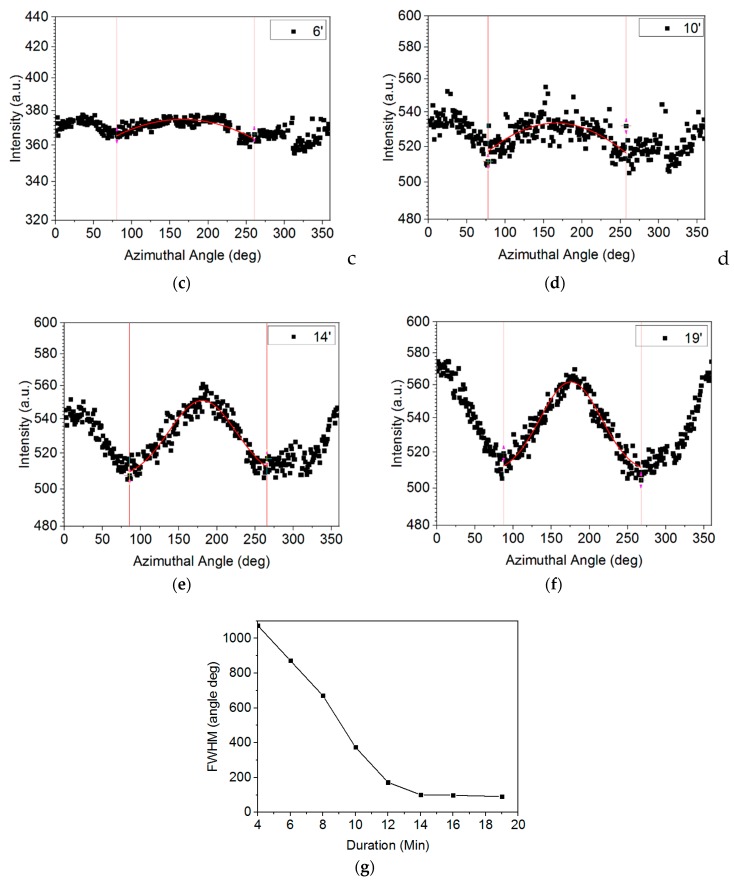
Illustration of the alignment and in-situ synchrotron SXAS results of magnetic nanoparticle doped hexagonal phase aligned with magnetic field. (**a**) Polydomain structure of magnetic nanoparticles doped hexagonal phase; (**b**) Magnetically-aligned hexagonal phase; (**c**–**f**) Corresponding azimuthal intensity of the primary Bragg peak for hexagonal phase aligned at 6, 10, 14, 19 min, respectively; and (**g**) FWHM of azimuthal intensity of in-situ measurement versus time of sample in the magnetic field.

The schematic representation of the relative position of LLCs and nanoparticles is presented in Figure 3a,b. Each hexagonal unit is composed of cylinders of very large aspect ratio, with relative infinite length compared to their diameters, formed by self-assembling surfactant DTAB in water. Before applying any magnetic field, hexagonal units orient randomly with doped magnetic nanoparticles along the adjacent unit (Figure 3a), while the hexagonal units with the doped magnetic nanoparticles alone were aligned along the direction of fixed magnetic field when a magnetic field was applied (Figure 3b).

In situ 2D SAXS patterns were acquired in the apparatus shown in Figure 1 to evaluate the alignment of the LLC nanostructures under a 60 mT magnetic field. A SAXS pattern was acquired at time intervals of 2~3 min between 1 s SAXS measurements. The effect of the magnetic field for different exposure duration on the alignment can be qualitatively evaluated by analysing the azimuthal intensity distribution around the (1 0) diffraction ring (Figure 3c–f) [32]. As the duration of the magnetic field on the sample increases, the scattering patterns become increasingly anisotropic. Initially, the azimuthal intensity distribution of the (1 0) reflection was found to be nearly isotropic (Figure 3c). This is the indicative of a random orientation in the bulk material. With an increasing duration of the magnetic field applied to the sample, an obvious peak appears in the azimuthal intensity profiles (Figure 3d–f).

To measure the alignment of the LLC nanostructure, a single Gaussian peak fitting was performed on the azimuthal profile [33]. The value of the full width at half maximum (FWHM) of the Gaussian is a measure of the alignment. The lower the FWHM, the higher the alignment. A Gaussian function is a function in the form of $f\left(x\right)=a\exp\left(-\frac{{\left(x-b\right)}^{2}}{2c^{2}}\right)+d$, where *a* is the height of the curve’s peak, *b* is the position of the peak centre, and *c* is a constant related to the width of the bell shape. $\mathrm{FWHM}=2\sqrt{2\ln2}c\approx 2.35482c$.

As the duration of the exposure to the magnetic field increased, the alignment of the hexagonal phase within the sample was found to become more pronounced with a narrowing Gaussian function corresponding to a decrease in FWHM (Figure 3g, located at top right corner of Figure 3). The alignment of the nanostructures within the magnetic field is illustrated from the profile of FWHM versus time. The alignment was very effective during the first period of 12 min as the FWHM reduced quickly from 1074 to 91 degrees followed by an abrupt change, and finally became relatively stable in the period between 14 and 19 min. The behaviours of magnetic nanoparticles and their interaction with the DTAB, PEGDA and water molecules all play important roles in the magnetic alignment, and are breakthrough points to figure out the mechanism of this alignment.

### 3.2. MD Simulation

In the present work, the interaction energy between magnetic nanoparticles and other molecules in the solution transferred from the surface of a magnetic nanoparticle, through intermolecular interactions of Fe_3_O_4_-water, Fe_3_O_4_-PEGDA, water-PEGDA, water-water, PEGDA-PEGDA, DTAB-water, and DTAB-PEGDA, to a structured DTAB cylinder was calculated through MD simulations. The intermolecular interaction energy calculated is decomposed into electrostatic and van der Waals interactions. Here, the head group of DTAB molecules is rigidly held by electrostatics and the tail group is flexible with the terminal -CH3 group being free.

In the model, a magnetite layer of 34.99 Å × 34.99 Å × 16.21 Å was constructed and the most morphologically predominant (001)-surface was chosen. As the surface was generated by cutting from the bulk structure, it was a polar surface. Therefore, the magnetite (001)-surface was stabilised through geometry optimization. The relaxed surface then interacted with isolated DTAB and PEGDA molecules, individually. 

Based on the results of the interactions of the magnetite surface with DTAB and PEGDA, a system consisting of a DTAB cylinder, 66 PEGDA molecules and 528 water molecules was generated and the molecular dynamics simulations were performed to calculate the energy transfer from magnetite particle to the DATA cylinder. The cylinder was formed by 60 DTAB molecules and has a radii of about 1.30 nm (distance from the outmost carbons to the axis of the cylinder along z-direction). PEGDA molecules of n = 4 were used. In the system the weight fractions of the DTAB, PEGDA and water are 0.42, 0.39, and 0.19, respectively, which is consistent with experiment (Section 3.1). Following a minimization of 2 ps of the system, a dynamic simulation of 50 ns was performed at 298 K and 1 atm. To model the effect of the movement of magnetite surface under a magnetic field, a force *P* (*P*_x_, 0, *P*_z_) with a constant magnitude, *P*, (*P*_x_^2^ + *P*_z_^2^ = *P*^2^) was imposed on the PEGDA and water atoms that locate on a layer normal to the x direction, i.e., the atoms meeting the coordinate condition of *x* < *x*_layer_, where *x*_layer_ was determined at each time step to maintain a constant force magnitude. The force direction was defined by the counterclockwise angle from the positive x-axis. The intermolecular interaction energy, *F*, of the DTAB with the PEGDA and water molecules under different imposed force directions was analysed based on the results of NVE molecular dynamics simulation of corresponding systems.

**Figure 4:**
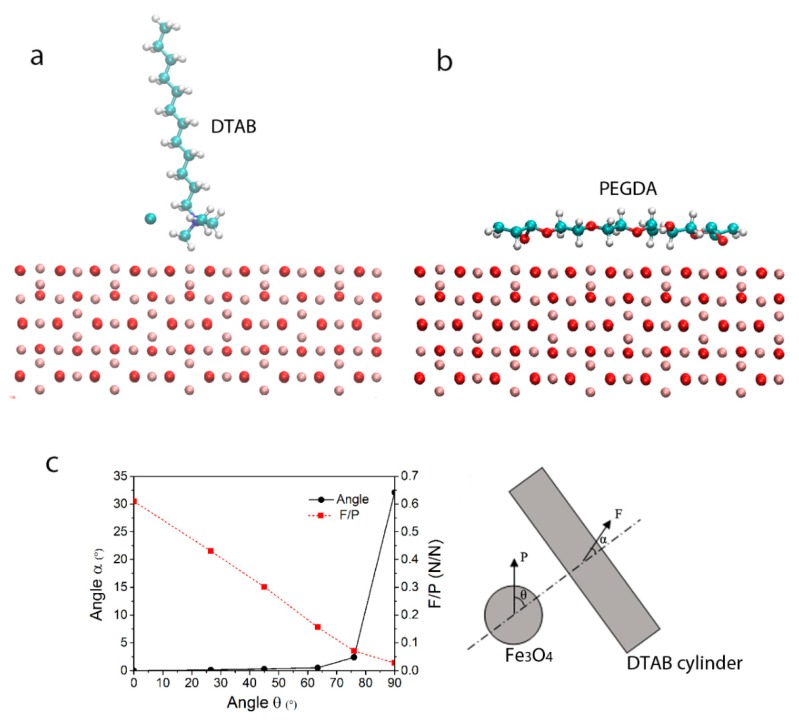
MD simulation results. The side view of the interactions of (**a**) the head group of DTAB (The DTAB body repulse to the Fe_3_O_4_ surface); and (**b**) PEGDA with (001) surface of magnetite. The Fe atoms in ochre, O in red, C in cyan, N in blue, H in white, and iodine ion in isolated cyan. (**c**) Magnitude and direction of force transfer from magnetite particle to the structured DTAB molecules. *θ* is the angle of magnetite particle force with a constant magnitude *P*. α is the angle of the force on DTAB cylinder, *F* (magnitude is *F*). Both angles are measured from the planes normal to the cylinder axis.

Figure 4a,b present the interactions between the surface of the magnetic nanoparticles with DTAB and PEGDA. The simulations show a repulsion of the surface of the magnetic nanoparticles to DTAB molecule. To compare the effect of different forces imposed on the magnetic nanoparticles, the change of DTAB cylindrical structure during the simulation should be confined, which was implemented by restricting the atoms of 12 alkyl groups of each DTAB molecule. Meanwhile, as the surface of the constructed cylinder is composed of the ammonium head-group (N and three of its connected carbon atoms) of the DTAB molecules, only the ammonium head-groups were considered in calculating the intermolecular interaction of DTAB-PEGDA and DTAB-water. The van der Waals and electric forces between atoms of the alkyl groups and the ammonium head-groups of the DTAB molecules were also ignored in the simulations by setting the depths of potential well and charges of carbon and hydrogen atoms in the 12 alkyl groups of DTAB molecules to zero. A weak adsorption of the head groups on the surface of nanoparticles was found (Figure 4a). The distance from nitrogen (N) atom of DTAB to the surface of magnetic nanoparticles (centre of mass of the first iron oxide layer) is 3.76 Å and the interaction energy calculated is −77.91 kJ/mol. Unlike the DTAB, the PEGDA showed a strong adsorption onto the surface of magnetic nanoparticles (Figure 4b) and the adsorption energy is −229.03 kJ/mol. The simulation results clearly show that the possibility of magnetic nanoparticles contacting with DTAB cylinder surface will be very low. On the contrary, most of the nanoparticles will be covered by the PEGDA molecules and stay in the solution of PEGDA and water, as illustrated in Figure 3a.

Based on above results, a system consisting of a DTAB cylinder, 66 PEGDA molecules and 528 water molecules was generated and the molecular dynamics simulations were performed to calculate the energy transfer from magnetite particle to DTAB cylinder. Figure 4c gives the relationship between the imposed force and the force transferred to the DTAB cylinder. When *θ* is less than about 65°, the *F*/*P* is above 0.15 and the angle α is less than 0.5°, indicating that the force transferred to the DTAB cylinders is effective in turning the cylinders to make it align in parallel to the magnetic field B. This step corresponds to the first stage of the LLC alignment before 12 min (Figure 3g). When *θ* is over 65°, the *F*/*P* is less than 0.15 and the angle *α* increases dramatically to 32° when *θ* reaches 90°, so only a small ratio of the force, P, transferred to the DTAB cylinders, and could not be big enough to make the LLC cylinders align further more dramatically (Figure 3g).

## 4. Retention of Aligned Nanostructure in Polymerized Films

**Figure 5:**
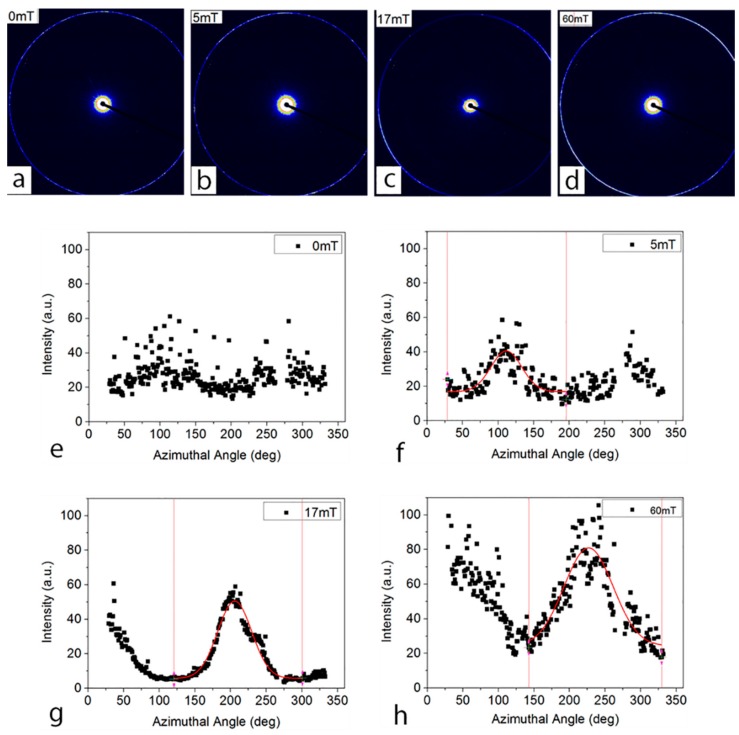
Structure alignment in the resulting polymer films. (**a**–**d**) Two dimensional SAXS patterns; and (**e**–**h**) Corresponding azimuthal intensity of the primary Bragg peak for polymer films templated with hexagonal LLC aligned under 0, 5, 17, and 60 mT, respectively.

The retention of alignment in the resulting polymerized membranes after UV irradiation was further investigated. Membranes were prepared by photo-polymerizing the prepared liquid samples after alignment under different magnetic field strengths. During the polymerization of the membrane, the polymer PEDGA molecules cross-linked around the aligned cylindrical nanostructure [34]. These prepared membranes were then scanned at the SAXS beamline to measure the retention of alignment. Both 2-D diffraction patterns and azimuthal intensity profiles are summarized to compare the alignment and retention of these membranes (Figure 5).

Figure 5a,e shows isotropic azimuthal intensity, which is in consistent with the fact that no magnetic field was applied to this sample before photo-polymerization. It is interesting to find that the alignment induced by the applied magnetic fields can be largely retained in the resulting polymerized membrane. The alignment was however found to be improved upon strengthening the magnetic field. The structures aligned under magnetic fields of 17 mT (Figure 5c,g) and 60 mT (Figure 5d,h) are much more anisotropic than the ones aligned at 5 mT (Figure 5b,f). This trend is consistent with previous research on the effect of magnetic field strength on the alignment of lamellar lyotropic mesophases in solution [13]. It was reported that a successful alignment of lyotropic lamellar systems under the magnetic field of 10 mT has been achieved [35], while no record of a lower magnetic field was found in the literature. Therefore, a low magnetic field at 5 mT might not be strong enough to generate macromolecular movement and, thus, alignment.

Interestingly, the FWHM of the membranes aligned at 17 mT is smaller than the one aligned at 60 mT despite the stronger magnetic field. To explain this result, the thickness of the samples was considered, and the result of thickness measurements is listed in Table 1. The membrane aligned at 17 mT is 10 µm thicker than the one aligned at 60 mT, this can keep the aligned nanostructure in a more stable equilibrium environment, receiving less thermal radiation during the photo-polymerization exposing to UV source. This can be further improved by controlling the thickness of the membranes to compare the alignment effect under different magnetic fields. Therefore, the alignment retained in the membrane depends on the magnetic field strength and the thickness of the membrane.

## 5. Conclusions

In conclusion, the alignment of hexagonal lyotropic liquid crystalline nanostructure (<5 nm) doped with magnetic nanoparticles, under magnetic fields has been achieved. The process of the magnetic alignment was for the first time acquired with in situ SAXS measurement in 2D diffraction pattern. The values of FWHM of the azimuthal intensity as a function of the duration present in magnetic field indicate that the magnetic alignment was proceeded in a reduced pace, and required about 20 min to reach a full alignment under the defined condition in this research. The alignment measured from SAXS is in good agreement with the observations from the molecular dynamics simulation. The aligned nanostructure was retained in the resulting polymerized membrane. The retention depends on the magnetic field strength, as well as the thickness of the membrane. Successful retention of the aligned cylindrical nanostructure after photo-polymerization of the membrane enables the nano-filtration/separation membrane potentially of high efficiency of selection and throughput. 

## Figures and Tables

**Table 1:** Comparison of FWHM and thickness between two polymerized membranes with alignment.

Sample	FWHM (°)	Thickness (µm)
17 mT	51.1	60
60 mT	81.5	50
